# Dynamic mechanical and thermal properties of clear aligners after thermoforming and aging

**DOI:** 10.1186/s40510-021-00362-8

**Published:** 2021-06-28

**Authors:** Kazem Dalaie, Seyyed Mostafa Fatemi, Samin Ghaffari

**Affiliations:** 1 Department of Orthodontics, School of Dentistry, Shahid Beheshti University of Medical Sciences, Daneshju Boulevard, Velenjak District, Tehran, Iran; 2 Department of Dental Biomaterials, School of Dentistry, Shahid Beheshti University of Medical Sciences, Tehran, Iran; 3 Dentofacial Deformities Research Center, Research Institute of Dental Sciences, Shahid Beheshti University of Medical Sciences, Tehran, Iran

**Keywords:** Clear aligner appliances, Dentistry, DMTA, Glass transition, Orthodontics, Polyethylene terephthalate glycol, Thermocycling, Thermoforming

## Abstract

**Background:**

Based on the role of properties of aligner materials on their efficiency, we aimed to assess their thermomechanical properties after thermoforming and simulated aging.

**Methods:**

In this experimental study, 96 samples of polyethylene terephthalate glycol (PETG) aligners (Duran and Erkodur) were prepared and divided to three groups: control (C), after thermoforming (T), after thermoforming and aging (TA). Thermoforming was done through 3D-printed molds, and aging was exerted by 200 thermal cycles after immersion in 37°C distilled water for 24h. Flexural modulus, hardness, glass transition temperature (Tg), elastic and viscous modulus, and loss factor were evaluated. Two-way ANOVA, T-independent, and Tukey HSD tests were done for statistical analysis and significance level was set to 0.05.

**Results:**

In both materials, flexural modulus decreased significantly after thermoforming, 88% in Duran and 70% in Erkodur, but did not change significantly after aging. After thermoforming, hardness decreased significantly in both materials (22% in Duran and 7.6% in Erkodur). Dynamic Tg was significantly lower in T and TA in both materials. At all temperatures (25, 37, 55°C) in Duran, the elastic modulus difference was only significant between C and TA, but in Erkodur, it decreased significantly in T, and there was no significant change after aging. Viscous modulus and loss factor showed the same change patterns at all temperatures. In both materials, they increased after thermoforming, but did not change significantly after aging.

**Conclusion:**

Thermoforming had more prominent role than aging in diminishing of thermomechanical properties. In general, Duran had greater thermomechanical stability than Erkodur.

## Background

Clear aligners, with widespread popularity due to their better comfort and esthetics especially among adult patients, now are an integral part of orthodontic treatments [1]. Clear aligner therapy (CAT) includes a series of removable aligners, which incremental tooth movements are planned by an allocated software [2]. At each aligner, which are supposed to be used for 2 weeks, linear tooth movements of 0.2 mm and rotational tooth movements of 1° are considered [3]. Despite the growing technology in this context, there are limitations to treat the malocclusions by CAT. Although leveling and alignment are well achieved with these appliances, some movements such as extrusion of anterior teeth, derotation of rounded teeth, and torque movements are accomplished by the aligners with lower predictability [4–6]. A recent update on the accuracy of different tooth movements with aligners showed that although the overall accuracy is improved, it is still 50% for total types of tooth movements, and same as before, rotation is the least accurate movement [7]. On the other hand, recently, it has been shown that aligners fail to correct overbite and anteroposterior discrepancies with high predictability, although they improve alignment and interproximal contacts in either class I or class II malocclusion [8]. Moreover, versus fixed appliances, they can result in comparable outcomes mostly in mild to moderate malocclusions [6].

In the search of why the effectiveness of aligners is lower than expected and how it can be improved, special attention should be paid to the materials of aligners and the process of their manufacture. Clear aligners are composed of thermoplastic resin polymers such as polyurethane (PU), polyethylene terephthalate (PET), polyethylene terephthalate glycol (PETG), and polyvinyl chloride [9]. Resin polymers are not inert, and they are prone to change in front of heat, humidity, constant forces, and saliva in the oral environment [10]. Hence, it seems logical that any weakening of aligner materials, either following the manufacturing process [11] or following exposure to the oral environment [12], would reduce their efficiency, and subsequently, less predictable tooth movements will occur. But, how this process occurs and how it can be limited have been less researched precisely in latter studies.

Previously, studies have confirmed the strong relation among the mechanical properties of aligner materials, such as hardness and elastic modulus, and the amount of force exerted by aligners [13, 14]. Therefore, any factor leading to considerable changes in the mechanical properties of aligners will cause changes in the force application system and the effectiveness of treatment. The effect of thermoforming [11, 15] and aging [10, 12, 16, 17] on the properties of different aligner materials have been evaluated previously; however, to the best of our knowledge, none of the studies have assessed and compared the effect of thermoforming and aging concomitantly. Moreover, controversies exist in literature in this context, and there is no consensus upon the influence of these factors.

Conclusively, we aimed to evaluate the dynamic mechanical and thermal properties of aligners after thermoforming and aging in a simulated oral environment. The null hypothesis was considered as mechanical and thermal properties of these materials do not change after thermoforming and in vitro aging.

## Materials and methods

### Preparation of the samples

In this experimental study, 2 types of aligner sheets composed of PETG, were utilized:

1. Duran (SCHEU-DENTAL GmbH, Iserlohn, Germany) with thickness of 1 mm.
2. Erkodur (Erkodent Erich Kopp GmbH, Pfalzgrafenweiler, Germany) with thickness of 0.8 mm.

Then, these sheets were divided into 3 groups:

1. Control group (C): untreated sheets without thermoforming or aging
2. Thermoforming group (T): sheets after applying thermoforming
3. Thermoforming and aging group (TA): sheets after applying thermoforming and aging

### Sample size calculation

Based on the fact that this study was experimental, probability of excluding a sample was set as zero. Also, considering α error equivalent to 0.05 and the power of the study equivalent to 0.8, the following formula was utilized to determine the sample size:

$$ {\displaystyle \begin{array}{l}n=\frac{{\left({Z}_{1-\alpha /2}+{Z}_{1-\beta}\right)}^2\left({SD}_1^2+{SD}_2^2\right)}{{\left({X}_1-{X}_2\right)}^2}\times \frac{1}{1-f}\\ {}\alpha =0.05\to {Z}_{1-\alpha /2}=1.96\\ {}\beta =0.2\to {Z}_{1-\beta }=1.83\\ {}f=0\end{array}} $$

Based on the study by Ryu et al. [11], which the flexural modulus of eCligner aligners was determined as 2313.1±112.2 MPa before thermoforming and 1897.4±169.6 MPa after thermoforming, the sample size was calculated as 4 for each group. In order to increase the power of sample size up to 25%, 5 samples were considered for each group. It is noteworthy to mention that only one sample was utilized for differential scanning calorimetry (DSC) in each group because this analysis was performed only to determine the glass transition temperature of samples for other relevant analysis.

Finally, a total of 96 samples, 48 for each material and 5 for each test, except 1 for DSC, were prepared and cut into preset sizes through ultrafine diamond discs (Jota, Rüthi, Switzerland) and interrupted swipes in order to prevent heat generation during this process.

### Thermoforming process

**Fig. 1:**
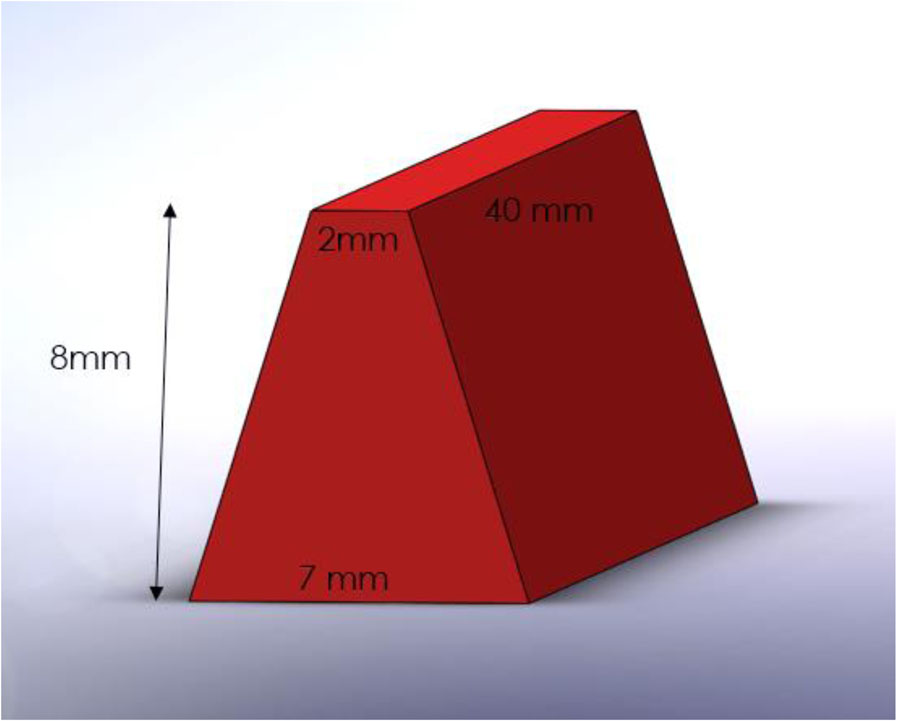
Designed 3D mold for thermoforming of aligner sheets

One of the objectives of this present study was to simulate the process of manufacturing of the aligners experimentally as much as possible. Due to the need to obtain samples with flat surfaces for different analysis, the conventional way of thermoforming of aligner sheets on printed dental models was not applicable. So, special 3D mold was designed by the Solidworks 3D software (Version 2019, Dassault Systèmes SolidWorks Corp., Waltham, MA, USA), which was similar to the mesiodistal surface of maxillary central incisor from the side view, as described by Ryu et al. previously [11]. The mentioned mold is illustrated in Fig. 1. Then, this mold was printed via fused deposition modeling (FDM) 3D printer by Cubicon Style (HyVISION, Seoul, South Korea) and continuous thermoplastic filaments, which commonly are used for printing of dental casts during aligner manufacture, with an accuracy of 100 microns and printing speed of 1cm/40–60min. At last, each layer was cured through 405 nm ultraviolet light within the printer.

**Fig. 2:**
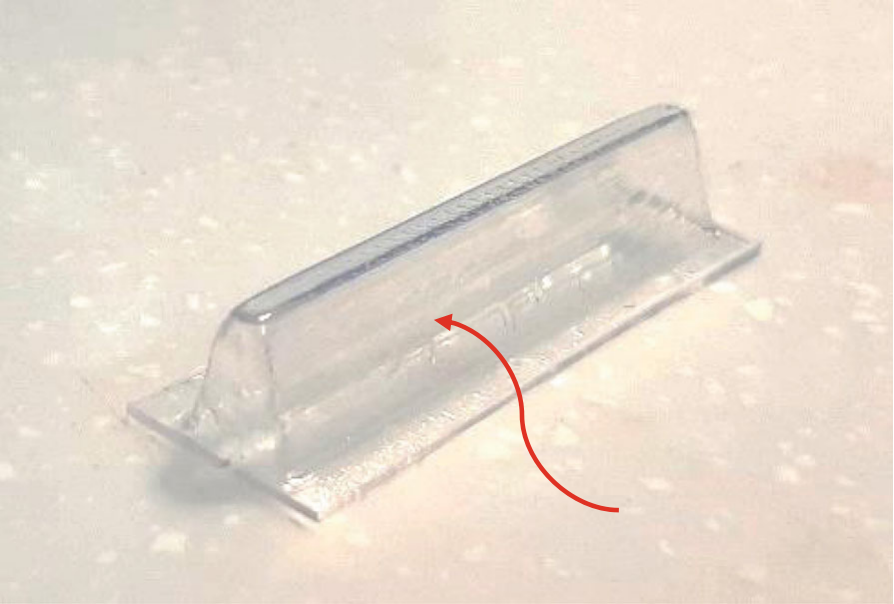
Thermoformed sheet after removing of mold from inside. The mentioned surfaces were used for specimens

After preparation of the molds, thermoforming was exerted by pressure former type thermoforming machine, minister S ® (SCHEU-DENTAL GmbH, Iserlohn, Germany). For thermoforming, pressure at 4bar/58psi, infrared heat at 160°C for 30s, and then cooling for 45s were applied. After this process, sloping side surfaces of the thermoformed sheets were utilized to prepare samples (Fig. 2).

### Aging process

In the third group, samples underwent an aging process in addition to the thermoforming. Thermocycling was used in this study to simulate temperature changes in oral environment. For this purpose, considering that patients should use each aligner at least 2 weeks and 22 h each day, 14 thermal cycles for each day and generally 200 cycles for 2 weeks were determined to maximize the simulation with oral conditions. Before thermocycling, samples were immersed in 37°C distilled water for 24 h, and then, thermal cycles including 5°C for 20s, 55°C for 20s, and a transfer time of 12s were exerted in distilled water via temperature aging machine (Thermocycler, Dorsa, Tehran, Iran) [17]. Then, they were stored inside distilted water in incubator (Pars Azma Co, Tehran, Iran) until the time of analysis.

In this study, thermal and mechanical evaluation of the aligners was exerted by the following analysis:

#### Three-point bending test

This test was performed to determine flexural modulus of the specimens. It was carried out by Universal Testing Machine (UTM- model Zwick/Roell®Z020, Zwick Roell, Genova, Italy). Loading was done with a rate of 1 mm/min with maximum deflection of 5mm [18]. Each specimen was prepared with a size of 4×20 mm and inserted on the fulcrums of machine at a distance of 2mm on each side (the unsupported length of the material between two fulcrums was 11 mm, considering the thickness of the support points) [18]. Based on the deflection of aligners during force application [11], 0.5 to 1 mm deflection of the specimens was considered for determination of flexural modulus, through the following formula:

$$ E=\frac{\left({F}_2-{F}_1\right){I}^3}{4b{h}^3\left({d}_2-{d}_1\right)} $$

where F_2_ is the loading force at 1 mm deflection (d_2_), F_1_ is the loading force at 0.5 mm deflection (d_1_), I is the distance between two fulcrums, b is the width of the specimen, and h is the height of the specimen.

#### Surface hardness test

Vickers hardness tester (Indentec, Zwick Roell, Genova, Italy) was utilized to determine the surface hardness. In this manner, specimens with a size of 9×13 mm were prepared, and at each specimen, 3 indentations were created through pyramidal diamond indenter under 10N force application for 10s [11]. Then, the diameters of the created squares were measured by a light microscope with ×40 magnification, and Vickers number or HV was calculated through the following formula:

$$ HV=1.854\ \frac{F}{d^2} $$

where F is the loaded force and d is the mean of the diameters of each indentation.

#### Differential scanning calorimetry (DSC)

This analysis was performed to determine the static glass transition temperature. It was carried out by Polyma ® (DSC214, Netzsch-Gerätebau GmbH, Germany), through application of increasing temperature from −70 up to 240°C with a rate of 10°C/min [19]. The resultant temperature was utilized to determine the temperature range in DMTA, which is described below.

#### Dynamic mechanical thermal analysis (DMTA)

This analysis was performed through DMTA machine (DMTA 242C, Netzsch-Gerätebau GmbH, Germany). This method, which is widely used for viscoelastic polymers, has been rarely used previously, for assessment of aligner materials. In this analysis, an oscillating stress or strain with incremental increase in temperature is applied, in order to determine the thermal and mechanical properties of the viscoelastic materials dynamically [20]. Applied temperature range was based on the glass transition temperature (Tg) of each material, previously determined by DSC, and it was Tg±50°C. This temperature range was applied with an increasing rate of 5°C/min. Also, the amount of dynamic force was 2N; static force was 0.01N, with frequency of 1Hz and range of 0.1%. The specimens for this analysis were prepared in dimensions of 4×20 mm. Finally, four variables were obtained as follows [21]:

- Elastic modulus or E′: It is also named as storage modulus and represents the amount of energy stored in solid or elastic phase.
- Viscous modulus or E″: It is also named as loss modulus and represents the amount of energy lost in liquid phase or irreversible deformation of the material.
- Loss factor or tanδ: it is also named as damping factor and is obtained from the ratio of the viscous modulus to the elastic modulus. This factor is an allocated scale of the loss of mechanical properties of material.

These three variables were evaluated and compared at four temperatures: 25°C (room temperature), 37°C (body temperature), 55°C (hot beverage temperature), and Tg.

iv) Dynamic Tg: this temperature, which shows the transition from glassy to rubbery state, was determined by the peak of the curve of tanδ [22].

### Statistical analysis

In order to evaluate the normal distribution of the resultant data and to evaluate the variance of the groups, one-sample Kolmogorov-Smirnov test and Levene test were applied, respectively. For synchronous comparison of the materials and groups, two-way ANOVA analysis was done, and in the case of significant interaction between them, T-independent test, one-way ANOVA analysis, and Tukey’s HSD test were utilized for subgroup analysis. Also, repeated measures ANOVA and Bonferroni test were exerted to compare variables in different temperatures. Significance level was set to 0.05, and analysis was done by the SPSS software (Version 20.0, SPSS Inc., Chicago, IL, USA).

## Results

According to the Kolmogorov-Smirnov test, all of the resultant data had normal distribution (*P*-value>0.05). So, parametric tests were utilized for statistical analysis of the data.

### Flexural modulus

**Fig. 3:**
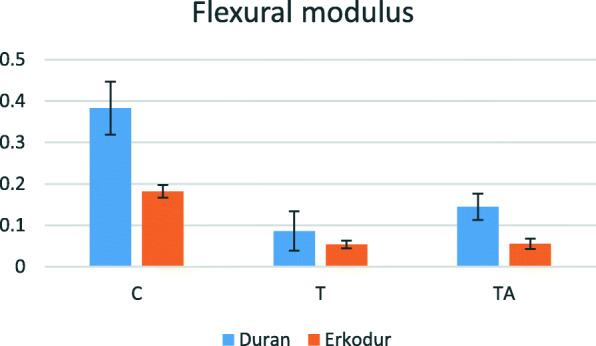
Changes in flexural modulus according to the material and groups

Mean of this variable in every group is presented in Table 1. According to the statistically significant interaction between the type of material (Duran and Erkodur) and groups (C, T, and TA) (P value<0.001), subgroup analysis was done. In both materials, flexural modulus decreased significantly after thermoforming (P value<0.001), 88% in Duran and 70% in Erkodur, but there was no significant difference between groups T and TA (P value=0.190 in Duran, P value=0.979 in Erkodur). Also, mean of flexural modulus was greater in Duran in comparison to Erkodur in groups C and TA (P value<0.001), but this difference was insignificant in group T (P value=0.173) (Fig. 3).

**Table 1:** Descriptive analysis of flexural modulus in MPa

Material	Group	Number	Mean	Standard deviation
Duran	C	5	0.3827	0.0639
	T	5	0.0859	0.0475
	TA	5	0.1445	0.0317
	Total	15	0.2044	0.1405
Erkodur	C	5	0.1816	0.0153
	T	5	0.0536	0.0090
	TA	5	0.0551	0.0127
	Total	15	0.0968	0.0631

### Hardness

**Fig. 4:**
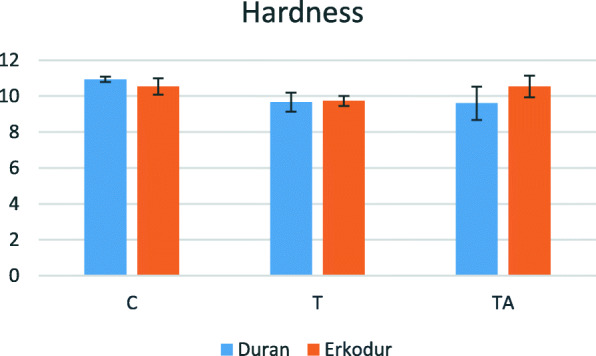
Changes in hardness according to the material and groups

Mean of this variable in every group is presented in Table 2. Considering statistically significant interaction between the type of material and different groups (P value=0.036), subgroup analysis was exerted. As seen in Fig. 4, in Duran, there was no significant difference between groups T and TA (P value=0.984), and both of them had significantly lower hardness than group C, 22% in both groups (respectively, P value=0.018 and 0.014). In Erkodur, the difference between groups C and TA was insignificant (P value=1.000), and both of them had greater hardness than group T (P value=0.045 in both groups). In other words, hardness decreased 7.6% after thermoforming. Comparing the two materials, in all groups, the difference between Duran and Erkodur was insignificant (P value>0.05).

**Table 2:** Descriptive analysis of hardness (hardness Vickers number)

Material	Group	Number	Mean	Standard deviation
Duran	C	5	10.9332	0.1493
	T	5	9.6664	0.527
	TA	5	9.5998	0.925
	Total	15	10.0664	0.8563
Erkodur	C	5	10.5332	0.4473
	T	5	9.7330	0.2791
	TA	5	10.5328	0.6056
	Total	15	10.2663	0.5801

### Static Tg

Resultant glass transition temperature in every group of each material is laid out in Table 3.

**Table 3:** Static glass transition temperature in each group of every material in terms of degrees Celsius

Material	Group		
	C	T	TA
Duran	76.3	73.5	82.1
Erkodur	76.6	70.7	68.2

### Dynamic Tg

**Fig. 5:**
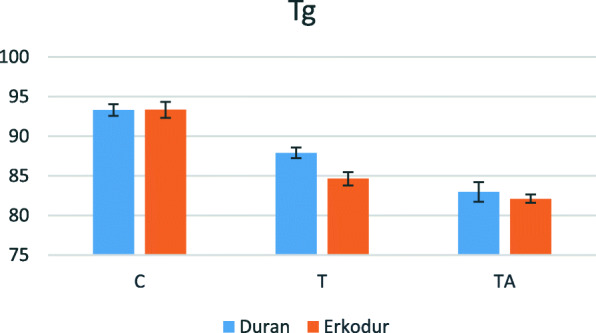
Changes in dynamic Tg according to the material and groups

Descriptive analysis of this variable is presented in Table 4. Based on statistically significant interaction between the type of material and groups (P value<0.001), subgroup analysis was done and showed that in both materials Tg significantly decreased in groups T and TA (P value≤0.001). Changes of this variable are shown in Fig. 5. Also, the difference of the mean of this variable was insignificant in group C (P value=0.945) and group TA (P value=0.181) between Duran and Erkodur. However, Duran had significantly greater dynamic Tg in comparison to Erkodur (P value<0.001) in group T.

**Table 4:** Descriptive analysis of dynamic Tg in terms of degrees Celsius

Material	Group	Number	Mean	Standard deviation
Duran	C	5	93.28	0.736
	T	5	87.88	0.669
	TA	5	82.96	1.228
	Total	15	88.04	4.444
Erkodur	C	5	93.32	1.011
	T	5	84.62	0.861
	TA	5	82.09	0.522
	Total	15	86.68	5.038

### Elastic modulus (E′)

**Fig. 6:**
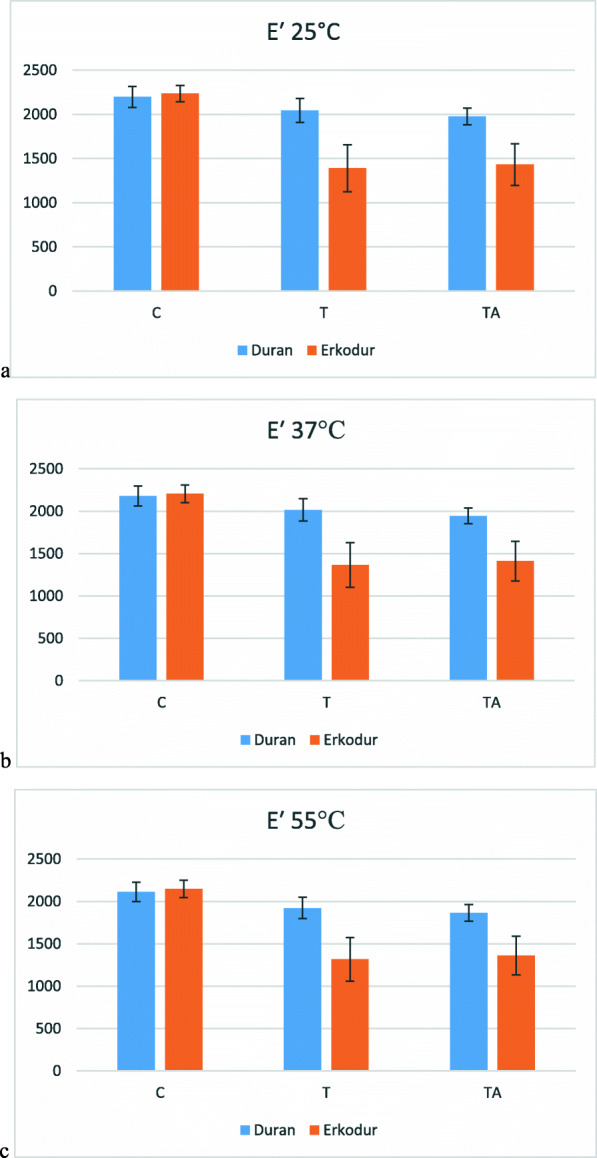
Changes in elastic modulus at **a** 25°C, **b** 37°C, **c** 55°C

Descriptive analysis of E′ in 4 temperatures is laid out in Table 5. Mean of E′ from 25°C up to glass transition temperature decreased significantly in Duran and Erkodur in all groups (C, T, TA) (P value<0.001). Changes of E′ at each temperature are shown in Fig. 6.

**Table 5:** Descriptive analysis of E′ at different temperatures

Material	Group		25°C	37°C	55°C	Tg
Duran	C	Mean	2198.155	2180.272	2111.009	1982.695
		SD	118.107	118.532	114.009	122.371
	T	Mean	2044.808	2015.000	1921.949	1746.128
		SD	136.756	132.229	125.618	110.811
	TA	Mean	1975.377	1943.784	1863.969	1064.145
		SD	95.370	92.961	98.656	181.881
Erkodur	C	Mean	2234.901	2205.153	2146.935	2031.713
		SD	93.769	105.677	102.303	99.738
	T	Mean	1389.595	1365.150	1316.964	1248.335
		SD	265.725	262.883	256.345	246.325
	TA	Mean	1432.395	1411.464	1363.356	1301.689
		SD	235.938	234.345	228.111	219.564

*SD* standard deviation

At 25°C, in Duran, E′ decreased after thermoforming about 7% (P value=0.141) and aging about 3.4% (P value=0.632); however, none of them was significant, although there was significant decrease between groups C and TA (P value=0.028). In Erkodur, there was no significant difference between groups T and TA (P value=0.946), and both of them had significantly lower E′ than group C (P value<0.001) (38% in T and 35.9 in TA). On the other hand, although the difference between Duran and Erkodur was insignificant in group C (P value=0.601), this variable had significantly greater mean in Duran in comparison to Erkodur in groups T and TA (P value=0.001 in both groups).

At 37°C, the changes in mean of E′ were same as 25°C. In Duran, only the difference between groups C and TA was significant (P value=0.018). Also, in Erkodur, the difference between groups T and TA was insignificant (P value=0.937), and both of them had significantly lower E′ than group C (P value<0.001 in both groups). Same as before, the difference between Duran and Erkodur was insignificant in group C (P value=0.933), but this variable had significantly greater mean in Duran in comparison to Erkodur in groups T and TA (P value=0.001 in both groups).

Also at 55°C, the changes in E′ were same as before. In Duran, only the difference between groups C and TA was significant (P value=0.012). In Erkodur, groups T and TA had no significant difference (P value=0.933), and both of them had significantly lower E′ than group C (P value<0.001 in both groups). Also, the difference between Duran and Erkodur was insignificant in group C (P value=0.614) and significant in groups T (P value=0.002) and TA (P value=0.001).

### Viscous modulus (E″)

**Fig. 7:**
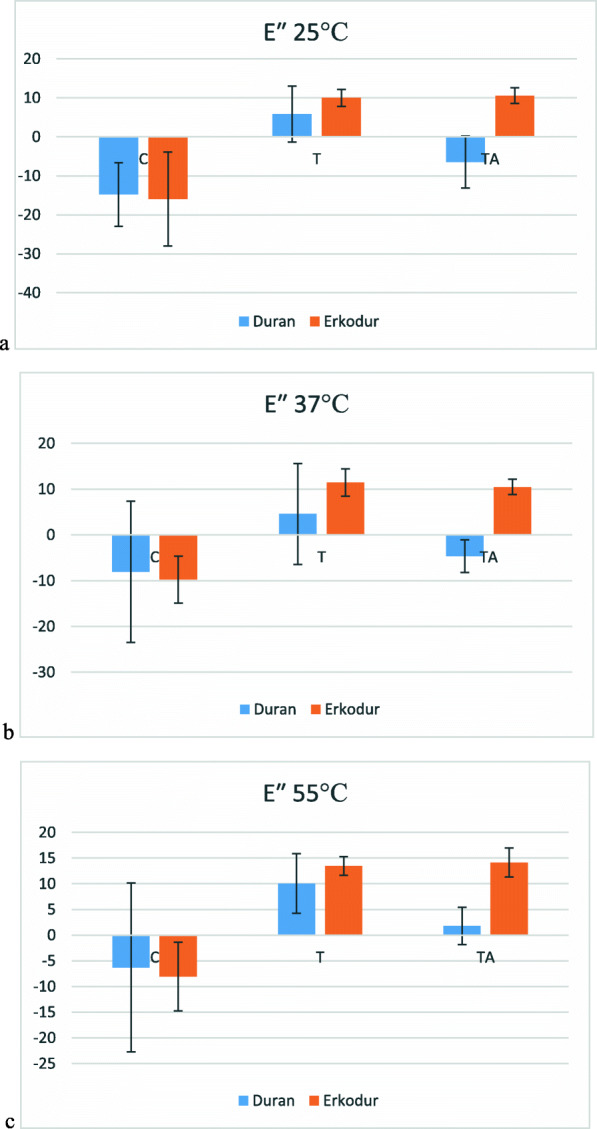
Changes in viscous modulus at **a** 25°C, **b** 37°C, **c** 55°C

Descriptive analysis of E″ is presented in Table 6. Mean of E″ significantly increased from 25°C up to Tg (P value<0.05) in every group of both materials, except group C in Erkodur (P value=0.297). Changes of E″ at each temperature are shown in Fig. 7.

**Table 6:** Descriptive analysis of E″ at different temperatures

Material	Group		25°C	37°C	55°C	Tg
Duran	C	Mean	−14.757	−8.067	−6.289	3.087
		SD	8.158	15.451	16.455	12.105
	T	Mean	5.835	4.584	10.061	32.351
		SD	7.193	11.043	5.827	6.418
	TA	Mean	−6.425	−4.639	1.797	197.960
		SD	6.670	3.584	3.634	39.487
Erkodur	C	Mean	−15.945	−9.737	−8.062	1.802
		SD	12.022	5.135	6.667	5.057
	T	Mean	10.008	11.461	13.493	18.710
		SD	2.213	2.970	1.819	2.092
	TA	Mean	10.557	10.474	14.120	20.531
		SD	2.005	1.661	2.823	4.066

*SD* standard deviation

At 25°C, in Duran, there was no significant difference between groups T and TA (*P*-value=0.053), but group T had significantly greater E″ than group C (P value=0.002), about 140%. In Erkodur, there was no significant difference between groups T and TA (P value=0.992), and both of them had significantly greater E″ than group C (P value<0.001 in both groups), 163% in T and 166% in TA. On the other hand, there was no significant difference between Duran and Erkodur in group C (P value=0.859) and group T (P value=0.250), but in group TA, the mean of E″ was significantly greater in Erkodur than Duran (P value=0.001).

At 37°C, in Duran, although group T had the highest mean of E″ between all groups, none of the differences between groups were significant (P value>0.05). In Erkodur, there was no significant difference between groups T and TA (P value=0.900), and both of them had significantly greater E″ than group C (P value<0.001 in both groups). Same as before, the difference between Duran and Erkodur was only significant in group TA (P value<0.001) and insignificant in groups C (P value=0.824) and T (P value=0.216).

At 55°C, same as before in Duran, none of the groups had significant difference with each other (P value>0.05), and in Erkodur, although there was no significant difference between group T and TA (P value=0.971), both of them had greater E″ than group C (P value<0.001). Also, the difference between Duran and Erkodur was only significant in group TA (P value<0.001) and insignificant in groups C (P value=0.244) and T (P value=0.829).

### Loss factor (tanδ)

**Fig. 8:**
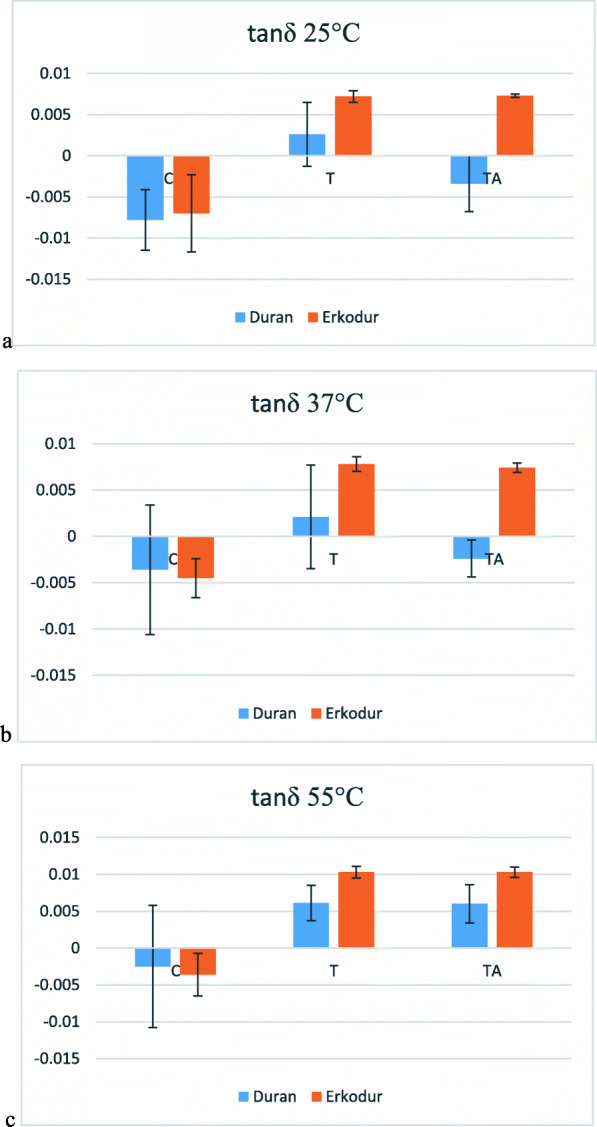
Changes in tanδ at **a** 25°C, **b** 37°C, **c** 55°C

Mean of this variable in each group at 4 temperatures is presented in Table 7. Mean of tanδ significantly increased from 25°C up to Tg (P value<0.050) except group C in Erkodur (P value=0.297). Changes in each group at each temperature are shown in Fig. 8.

**Table 7:** Descriptive analysis of tanδ at different temperatures

Material	Group		25°C	37°C	55°C	Tg
Duran	C	Mean	−0.0078	−0.0036	−0.0025	0.0018
		SD	0.0037	0.0070	0.0083	0.0066
	T	Mean	0.0026	0.0021	0.0061	0.0182
		SD	0.0039	0.0056	0.0024	0.0025
	TA	Mean	−0.0034	−0.0024	0.006	0.2007
		SD	0.0034	0.0020	0.0026	0.0740
Erkodur	C	Mean	−0.0070	−0.0045	−0.0036	0.0010
		SD	0.0047	0.0021	0.0029	0.0026
	T	Mean	0.0072	0.0078	0.0103	0.0153
		SD	0.0007	0.0008	0.0008	0.0021
	TA	Mean	0.0073	0.0074	0.0103	0.0157
		SD	0.0002	0.0005	0.0007	0.0011

At 25°C, in Duran, group T had greater tanδ in comparison to group C (P value=0.002) and group TA (P value=0.061). Also, the difference between group T and TA was insignificant (P value=0.188). In Erkodur, there was no significant difference between groups T and TA (P value=0.996), and both of them had significantly greater tanδ in comparison to group C (P value<0.001), 203% in T and 204% in TA. The difference between Duran and Erkodur was insignificant in group C (P value=0.786) and significant in groups T (P value=0.032) and TA (P value<0.001).

At 37°C, there was no significant difference between groups in Duran (P value>0.05). In Erkodur, there was no significant difference between groups T and TA (P value=0.867), and both of them had significantly greater tanδ in comparison to group C (P value<0.001). Also, the difference between Duran and Erkodur was only significant in group TA (P value<0.001) and insignificant in groups C (P value=0.793) and T (P value=0.053).

In 55°C, same as before, the differences between groups in Duran was insignificant (P value>0.05). Also, in Erkodur, the difference between groups T and TA was insignificant (P value=1.000), and both of them had greater mean of tanδ in comparison to group C (P value<0.001). On the other hand, mean of tanδ had no significant difference in group C between Duran and Erkodur (P value=0.794), but this difference was significant in group T (P value=0.006) and group TA (P value<0.001).

## Discussion

In this study, different thermal and mechanical properties of two aligner materials, Duran and Erkodur, were evaluated through experimental methods after exerting thermoforming and in vitro aging. Given that there was a change in all parameters, the null hypothesis was rejected.

Duran and Erkodur aligners are both made of PETG, but they have different thicknesses, which is 1 mm in Duran and 0.8 mm in Erkodur. So, it made this possible to compare the different thicknesses of same composition in addition to evaluating two different materials. In general, Duran had greater thermal and mechanical properties than Erkodur, which can be attributed to its greater thickness.

In both materials, flexural modulus significantly decreased after both thermoforming and thermocycling processes compared to the control group, although only thermoforming caused a significant decrease in flexural modulus, and the changes after thermocycling were not significant in both materials. In other words, thermoforming has a more dominant role to weaken the aligner materials in comparison to in vitro aging, simulating the intraoral aging of aligners for 2 weeks. Considering the significant decrease in the thickness of aligners after thermoforming [11, 15], the reduction in flexural modulus seems justifiable.

In another study by Ryu et al. [11], it was observed that Duran aligners with 0.75-mm thickness showed an increase in flexural modulus after thermoforming, but the ones with 1-mm thickness showed a decrease after this process. The Duran material that was used in the present study also had 1-mm thickness. Furthermore, this study showed that flexural modulus decreases with increasing material thickness; however, in our study Duran, which had greater thickness than Erkodur, showed higher flexural modulus in all groups.

Elkholy et al. [18] evaluated different methods of three-point bending test for measuring flexural modulus for PETG aligners. They suggested that, given that aligners apply forces on teeth through very small deflections and with small distances between force application points on tooth surfaces, the lesser distance between two supports in three point bending test is preferred. In the present study, 11 mm was set as the distance between two supports in UTM, almost similar to the distance between two points of force application in aligners. Also, they have suggested that cracking in the material during the test leads to distorted results. In our study, none of the specimens had macroscopic cracks after the test. Furthermore, they observed a significant decrease in flexural modulus in all groups after thermoforming which is consonant with the present study results.

Another mechanical property evaluated in our study, was Vickers hardness. A previous study by Kohda et al. [13] indicated that there is a strong relation between the hardness of different aligners (Duran, Erkodur, and Hardcast) and the amount of applied force by them. So, changes in hardness can properly indicate the changes in applied force and consequently the efficiency of aligner therapy. In this study, in both materials, hardness significantly decreased after thermoforming. However, a previous study [11] showed that thermoforming does not influence the hardness of Duran.

There is controversy about the effect of aging on hardness in previous studies. Bradley et al. [12] showed a decrease in hardness of Invisalign after using by patients for 44±15 days, but Schuster et al. [10] showed an increase in hardness of the same aligners after 14 days of intraoral aging. It must be considered that the composition of Invisalign and utilized aligners for the present study are different. In the current study, thermocycling did not cause significant change in the hardness of Duran, but caused a significant increase in hardness of Erkodur. This increase in the hardness may be attributed to changes in crystal and amorphous structures or release of plasticizers after exerting intermittent thermal cycles, which should be evaluated more accurately in future studies. Although previous studies have shown no changes in chemical structures of Invisalign aligners after intraoral aging [12] and also in vitro aging [10], to the best of our knowledge, no study has evaluated the changes in chemical structures of PETG aligners. Same as before, thermoforming had a more obvious effect on hardness rather than thermocycling.

On the other hand, Iijima et al. [23] claimed that the hardness of different aligners, such as Duran, does not change significantly after 500 thermal cycles but significantly decreases after 2500 cycles. Since every aligner is frequently used about 2 weeks, we exerted 200 thermal cycles, and similar to the mentioned study, it did not lead to changes in the hardness of Duran.

In the present study, glass transition temperature of aligners was analyzed by both DSC and DMTA. According to DSC, Tg of untreated aligner sheets was 76.3°C in Duran and 76.6°C in Erkodur. Furthermore, previously, this temperature was measured at about 80°C in pure PETG [24], 75.3°C in Duran [23], and 77.2°C in Erkodur [19], which are in line with the results of our study. In this study, the dynamic Tg evaluated by DMTA, significantly decreased after thermoforming and also thermocycling in both materials, indicating the attenuation of thermomechanical properties after these processes.

It was confirmed that the influential factor on the exerted force by aligners is their Tg and not their crystal structure, and glass transition temperature can be an appropriate representative of the efficiency of aligners. Also, they showed that Duran aligners have higher mechanical stability due to their higher Tg than other aligner materials [23], which consequently leads to its higher stability at the maximum rate of increase in oral temperature after consumption of a warm drink (57°C) [25]. Similarly, in the present study, both materials had the same dynamic Tg in the control group, but it was higher in Duran than Erkodur after thermoforming and aging, which confirms the results of the mentioned study.

Now it is well-known that DMTA determines Tg with higher sensitivity and reliability compared to DSC [22] and the resultant temperature has higher values in the first method [19, 26]. In the present study as well, the resultant Tg by DMTA had higher values than DSC in both materials and all groups.

In the current study, elastic modulus was assessed by DMTA. Previously, tensile test or three-point bending test were utilized to determine elastic modulus. In these conventional methods, loading is applied constantly, but in DMTA besides sinusoidal loading, a gradual increase in temperature is exerted, so the thermomechanical properties of viscoelastic materials are evaluated with greater accuracy [21]. Formerly, the importance of assessment of elastic modulus in aligner materials have been indicated, that there is a strong relation between exerted force by aligners and their elastic modulus [13]. Also, elastic modulus, or equivalently storage modulus, stands for the amount of energy that is stored inside the viscoelastic material [21]. So, we can boldly say that the amount of energy remains stored inside the aligner will be then expressed as the force exerted on the teeth.

This analysis showed that elastic modulus decreases significantly by increasing temperature and the intensity of this decrement is greater at higher temperatures, which emphasizes diminished mechanical properties at higher temperatures. In Duran at all three temperatures (25, 37, and 55°C), thermoforming and thermocycling individually did not cause a significant decrease in elastic modulus, but their cumulative effect was significant, which caused about 10.2% decrease in elastic modulus. However, in Erkodur at all three temperatures, elastic modulus decreased significantly after thermoforming and did not change significantly after thermocycling. Therefore, when the aligner is placed over the teeth and starts force application, at constant strain the amount of stress they exert will be less than expected. Two materials had similar elastic modulus in the control group, but then it decreased greater in Erkodur, which reemphasizes the greater mechanical properties of Duran.

In this study, the mean of elastic modulus in untreated sheets at 25°C was 2198.1±118.1MPa in Duran and 2234.9±93.7MPa in Erkodur. A previous study by Daniele et al. as well [19] defined this variable as 2160 to 2430MPa in PETG aligners at 25°C. Also, another study by Ryu et al. [11] observed attenuation of elastic modulus after thermoforming, which was measured by tensile test and was in accordance to our results. On the other hand, Ryokawa et al. [15] observed an increase in elastic modulus of Duran, measured via tensile test, after thermoforming and also after immersion in 37°C distilled water. The difference can be attributed to various evaluation methods of elastic modulus and also simulation of intraoral aging.

Previously, Ihssen et al. [17] evaluated elastic modulus of PETG aligners by tensile test at 22 and 37°C and assessed the effect of thermocycling with 1000 thermal cycles. They observed a significant decrease in elastic modulus after thermocycling; however, in our study, it did not change significantly after 200 cycles. Furthermore, they observed a lower elastic modulus at 37°C, which is consonant with the results of the current study.

Also, viscous modulus and loss factor were evaluated by DMTA. Both of them represent the loss of thermomechanical properties. In other words, the higher the loss factor, the less mechanical stability [21]. Both of these factors had a similar pattern of changes at 25, 37, and 55°C. In both materials, they increased with increasing temperature, and the intensity of this increase was greater at higher temperatures. In both materials and at all three temperatures, these factors increased after thermoforming but did not change significantly after aging, again indicating the prominent role of thermoforming. Also, after thermoforming and especially after thermocycling Erkodur had a higher viscous modulus and loss factor than Duran.

The present report evaluated the influence of aging on clear aligners. However, it should be taken into account that wear [27] or brushing [28] can alter the surface characteristics of the orthodontic materials. Therefore, further studies are needed in order to also consider the possible effects of other unexplored variables.

Generally, considering the normal distribution of data and remarkably low values of standard deviation, DMTA can favorably be utilized for evaluating the different aligner materials in future studies.

The limitations of our study were that intraoral aging was simulated only by thermal cycles, and fatigue caused by loading of occlusal forces was not considered. Also, only aligners composed of PETG were evaluated, and thermomechanical properties of different aligner materials were not compared. It is suggested that in future researches, different aligner compositions such as polyurethane, polyethylene terephthalate, or copolyester and also their different thicknesses be evaluated and compared through DMTA. It is also implicated to evaluate changes in chemical and crystal structures of aligners besides their thermal and mechanical properties, so the cause of the attenuation of these properties after different processes might be understood in basic structures of these materials and get improved in the future.

## Conclusion

- Generally, Duran has higher thermal and mechanical stability than Erkodur and can be utilized for clear aligner treatments with higher efficiency.
- In both materials, thermoforming was the main factor in diminishing of thermal and mechanical properties, and simulated aging did not lead to significant changes in most properties.
- In both materials, all variables, except viscous modulus and loss factor, decreased after thermoforming, but after aging, some properties such as hardness of Erkodur and to a lesser extent flexural modulus of Duran increased and other variables decreased.
- Due to significant increase in the loss factor with increasing temperature, keeping aligners in room temperature and avoiding hot liquids while using them are emphasized.

## Data Availability

The datasets used and/or analyzed during the current study are available from the corresponding author on reasonable request. Also, the datasets supporting the conclusions of this article are included within the article.
